# Risk of Allergic Rhinitis, Allergic Conjunctivitis, and Eczema in Children Born to Mothers with Gum Inflammation during Pregnancy

**DOI:** 10.1371/journal.pone.0156185

**Published:** 2016-05-25

**Authors:** Vivian Chia-Rong Hsieh, Chin-Chen Liu, Yu-Chen Hsiao, Trong-Neng Wu

**Affiliations:** 1 Department of Health Services Administration, College of Public Health, China Medical University, Taichung, Taiwan; 2 Department of Family Medicine, E-Da Hospital, Kaohsiung, Taiwan; 3 Center for General Education, Asia University, Wufeng, Taiwan; 4 Department of Nursing, College of Medicine and Nursing, Hungkuang University, Taichung, Taiwan; 5 Division of Environmental Health and Occupational Medicine, National Health Research Institutes, Zhunan, Taiwan; 6 Department of Healthcare Administration, Asia University, Wufeng, Taiwan; Tongji Medical College, CHINA

## Abstract

**Purpose:**

Despite links between maternal and child health status, evidence on the association between gum infection in pregnant mothers and childhood allergies is scarce. We aim to evaluate the risk of developing allergy in children born to periodontal mothers in a nationwide study.

**Methods:**

We conducted a 9-year population-based, retrospective cohort study using Taiwan’s National Health Insurance database. A study cohort of 42,217 newborns born to mothers with periodontal disease during pregnancy was identified in 2001 and matched with 42,334 babies born to mothers without any infection (control) by mother’s age at delivery and baby sex. With a follow-up period from 2001 to 2010, we observed the incidence of allergic rhinitis (AR), allergic conjunctivitis (AC), and eczema in these children. Cox proportional hazards regression models were performed with premature deaths as competing risk for the estimation of allergic disease risks.

**Results:**

Nine-year cumulative incidences were the highest among children born to periodontal mothers; they reached 46.8%, 24.2%, and 40.4% (vs. 39.5%, 18.3% and 34.8% in control) for AR, AC, and eczema, respectively. Our results showed moderately increased risks for the allergies in children born to periodontal mothers relative to their matched non-inflammatory control (adjusted HRs: 1.17, 95% CI: 1.15–1.20; 1.27, 1.24–1.31; 1.14, 1.12–1.17, respectively). Because the impact of food consumption and living environment cannot be considered using insurance data, we attempted to control it by adjusting for parental income and mother’s residential area.

**Conclusions:**

Overall cumulative incidence and risks of children born to periodontal mothers for AR, AC, and eczema are significantly higher than those born to non-inflammatory mothers. Gum infection in women during pregnancy is an independent risk factor for allergic diseases in children, thus its intergenerational consequences should be considered in gestational care.

## Introduction

Prevalence of allergic diseases is progressively increasing in children of Asian communities [1,2]. In Taiwan, allergic sensitization is very common as its cities have one of the most populous living conditions in the world. For this reason, childhood allergies have become a major public health issue.

The International Study of Asthma and Allergies in Childhood (ISAAC) has mapped out a significant variation and increase in the prevalence of childhood allergies across many countries [3]. Eczema, or atopic dermatitis, is the first to manifest in early infancy. It is an immunoglobulin E (IgE)-mediated, allergic skin disease with a complex etiology that is accompanied by superficial inflammation and itchy rashes. It is believed to be partly influenced by perinatal exposures and parental health problems [4,5]. Allergic rhinitis (AR) is a very prevalent atopic condition characterized by sensitized nasal mucosa and airway obstruction that is found mostly in boys and girls up to 10 years of age. It shares a common pathogenesis with eczema, and its response is initiated through contact with allergens in the air which causes loss of smell and affects sleep in children [6]. Allergic conjunctivitis (AC) is a type of ocular inflammation that is also common in children which causes redness and swelling of the eyes, with no distinction between boys and girls. Its pathophysiology involves a type I IgE-mediated immune reaction triggered by allergens contacting surface of the eye [7]. Altogether, these three conditions are rarely life threatening, but they can exert profound lifelong effect on the quality of life and the development of children.

Because periodontitis induces elevated inflammatory mediators and is also prevalent in young females, emerging studies have begun to scrutinize the impact of maternal infections and gingival inflammation on the health of their offspring [8,9]. Periodontitis is a chronic, bacteria-induced oral infection that causes gum-bleeding and detachment of tooth, while gingivitis is a milder form of gum inflammation that is also caused by bacteria. Gingivitis and periodontitis are collectively known as periodontal disease. This condition can trigger antibody responses against oral pathogens that lead to inflammation and destruction of periodontal tissues [10]. Periodontitis in pregnant females has not only been linked with poor neonatal outcomes [11], its pathogens are also believed to be capable of entering the circulation and generate prenatal or *in utero* sensitization [12,13]. This disease is characterized by a destructive inflammatory process which causes ulcerated gingival pockets that can give off bacteria and pro-inflammatory cytokines (i.e. IL-1, IL-6) into the systemic circulation [14]. It is possible that these cross the placenta into fetal circulation and ammonitic fluid and cause a surge in Th2 cytokines and decline in IFN-gamma secretions in children, directly impacting their cytokine profile that favor allergy development [15]. In other words, maternal profile of Th1 and Th2 immune responses during pregnancy would lead to transplacental transfer of factors that influence the development of immunologic responses in fetuses such as IgE-induced sensitization. As of now, cord-blood IgE (CB-IgE) is the most documented predictor for early sensitization [16–19]. The role of maternal allergen exposure during pregnancy in early life sensitization has further been demonstrated in animals [20]. Although the prevalence of periodontitis in young Taiwanese females has not been precisely estimated, Taiwan’s 2005 national health statistics indicate that around 31% of the population aged 16–64 years received periodontal and/or gingival treatment [11]. A community-based study revealed a prevalence of over 90% in its 35–44 years subject sample [21].

The purpose of the present study is to illustrate the relationship between maternal periodontal diseases during pregnancy and the early life onset of AR, AC, and eczema. We hypothesize that gum inflammation in mothers during pregnancy would increase the risk of developing these allergic diseases in their children.

## Materials and Methods

### Study design and data source

This was a retrospective cohort study with 2 groups of inflammatory, pregnant mothers and 1 control group of non-inflammatory, pregnant mothers. We designed a nine-year study from 2001 to 2010 to assess the development of AR, AC, and eczema in children born to mothers who were primarily diagnosed with periodontal disease (periodontitis and gingivitis) during pregnancy. We used claims-based information from the compulsory National Health Insurance (NHI), which forms a comprehensive population-based database managed and provided by The Collaboration Center of Health Information Application (CCHIA), Ministry of Health and Welfare in Taiwan. This research database contains health care data of over 99% of Taiwan’s population including enrollment registry, dates of outpatient visits and inpatient stays, diagnostic codes, prescription orders and related cost items ranging from laboratory tests to surgical procedures. The extensiveness and quality of this database can be supported by numerous pediatric and immunoepidemiology studies found in existing literature [11,22–24].

### Ethics statement

Because the Taiwan CCHIA-NHI database contains entirely of encrypted secondary data released to the public for research purposes, this study was exempted from a full review by the ethics review committee at the China Medical University and Hospital.

### Subject selection

**Fig 1:**
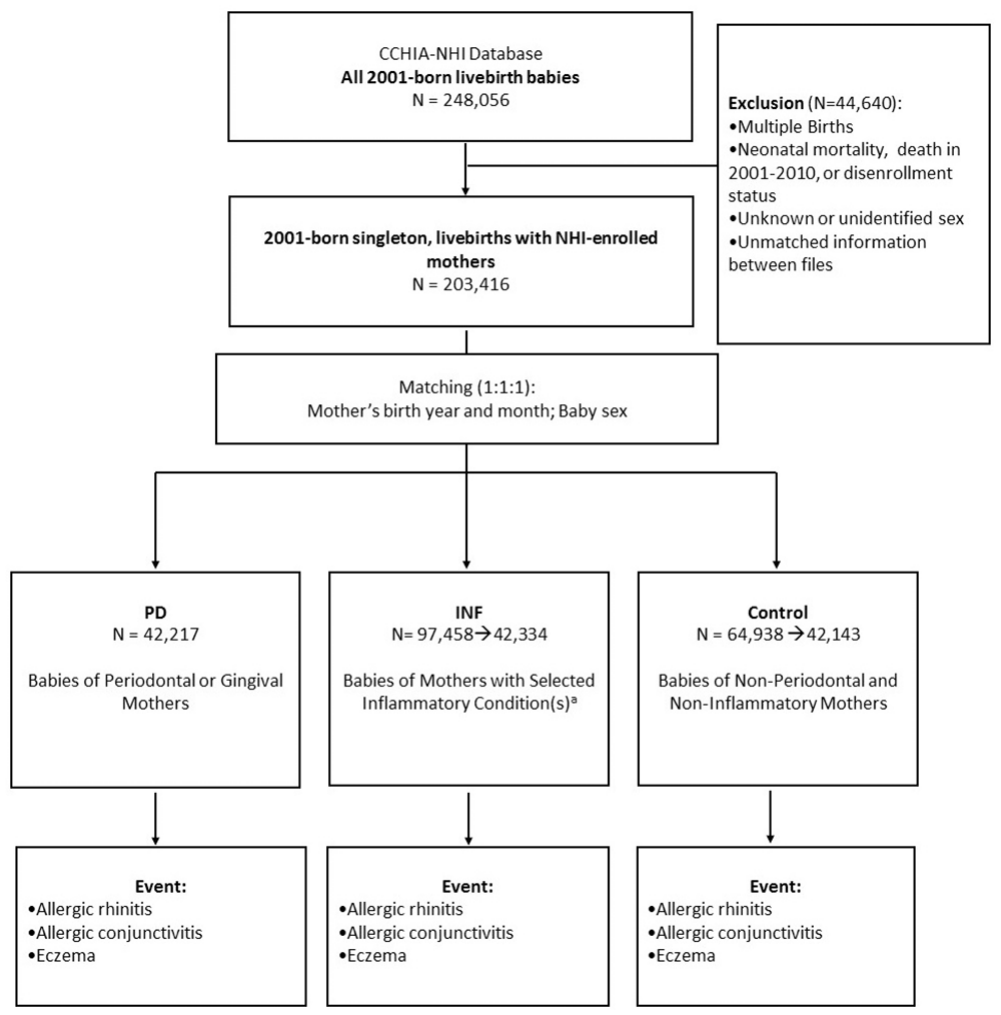
Flow diagram of subject selection and event definition. ^a^ Selected inflammatory conditions include urinary tract infection, bacterial vaginosis, infections of genitourinary tract, infections of kidney, inflammatory disease of cervix, vagina and vulva, as well as periodontitis and gingivitis.

We identified 248,056 livebirth babies born in 2001 from the CCHIA-NHI database. A total of 203,416 singleton babies were screened after exclusion of multiple births, neonatal mortality, lack of continuous enrollment data between 2001–2010, and unidentifiable sex or unmatched information between files (Fig 1). We first compiled our study cohort (PD) consisting of babies born to mothers who were diagnosed with periodontal disease for at least 1 episode during their pregnancy (n = 42,217). It was matched (1:1) with the control cohort which had babies born to mothers who were free of periodontal disease or other infections in pregnancy (n = 42,143). Because mother’s older age and boys are found to have an increased risk of allergic diseases throughout childhood [25], our matching criteria included baby sex and mother’s birth year and month. We considered additional inflammatory infections by identifying a second comparative cohort (INF) which was matched (1:1) to the control cohort in the similar manner; the INF cohort was comprised of babies born to mothers with selected inflammatory conditions (with at least 1 outpatient or inpatient visit) including urinary tract infection, bacterial vaginosis, infections of genitourinary tract, infections of kidney, inflammatory disease of cervix, vagina and vulva, as well as periodontitis and gingivitis during pregnancy (n = 42,334) (Fig 1). The INF and control cohorts were randomly selected from the populations (i.e., N = 97,458 for INF; N = 64,938 for control) to prevent possible exceeding power.

### Gum inflammation during pregnancy

We are primarily concerned with the potential effect of exposure to mothers’ oral bacterial infections by babies during their fetal development. Thus, we traced the period of their pregnancy from the database and specifically identified dental visits with periodontal disease-related treatments using International Classification of Diseases, Ninth Revision, Clinical Modification (ICD-9-CM) codes (S1 Table). Women detected with any diagnostic code for periodontitis or gingivitis during this period are categorized as periodontal mothers. Characterizing other selected inflammatory conditions like urinary tract infection was done in a similar manner (S1 Table).

### Identification of allergic rhinitis, allergic conjunctivitis, and eczema cases

All selected babies were followed since birth until event (allergic rhinitis (AR) ICD-9-CM 477, allergic conjunctivitis (AC) ICD-9-CM 372.14, or eczema ICD-9-CM 692), the end of 2010 (i.e., 31 December 2010), or disenrollment from the NHI. Each event was confirmed after identification of three consecutive diagnoses with the same ICD-9-CM code to rule out arbitrary diagnosis (S2 Table). For each confirmed event, the first date with such diagnosis is considered the date of event.

### Pregnancy history and comorbidities

Since the etiology of child health is multifaceted, we tried to control for factors originated from mothers to the prenatal and infancy period. Maternal comorbidities up to one year prior to delivery were identified. First, indication of high-risk pregnancy related conditions including history of abortions, poor obstetric history, and poor reproductive history was considered a potential sources of bias. Second, we detected systemic conditions including diabetes mellitus, hypertension and pre-eclampsia, chronic renal disease, chronic disease of the lung, smoking, alcohol, drug and substance abuse, edema, and anemia. Lastly, diseases localized to reproductive tract that may influence fetal health were also accounted for, including antepartum hemorrhage, chorioamnionitis, amniotic sac, cervical incompetence, and structural abnormality in uterus, cervix, vagina, or vulva (S3 Table).

### Statistical methods

Distributions of baseline characteristics for the 3 study cohorts and maternal comorbidities are described in Tables 1 and 2 using Chi-square tests and ANOVA. Frequency of gum inflammation and selected infections was compared between different cohorts. Kaplan-Meier curves are used to illustrate the cumulative incidence of each of the three allergic conditions. Log-rank tests are later performed to assess the statistical significance of their overall differences.

**Table 1:** Baseline socio-demographic attributes and infections of the 2001-born singleton matched cohorts.

	PD		INF		Control		*p*-value
	N = 42,217		N = 42,334		N = 42,143		
	No.	%	No.	%	No.	%	
**Sex**							0.990
Female	20134	47.69	20182	47.67	20107	47.71	
Male	22083	52.31	22152	52.33	22036	52.29	
**Mother's residential area**							<0.001
North	18893	44.75	18486	43.67	18740	44.74	
Center	12306	29.15	11651	27.52	11415	27.09	
South	10051	23.81	11061	26.13	10602	25.16	
East	752	1.78	899	2.12	1186	2.81	
Remote islands	215	0.51	237	0.56	200	0.47	
**Maternal age at delivery (years)**							0.9991
<20	942	2.23	942	2.23	935	2.22	
20–29	25164	59.61	25314	59.80	25152	59.68	
30–39	15740	37.28	15708	37.10	15690	37.23	
≧40	371	0.88	370	0.87	366	0.87	
Mean ± SD (years)	28.6	4.6	28.6	4.6	28.6	4.6	1.00
**Parental income (NTD)**							<0.001
Q5: 38201–87600	9043	21.45	9374	22.17	10004	23.79	
Q4: 26401–38200	9823	23.30	10675	25.25	10277	24.44	
Q3: 19201–26400	6948	16.48	6957	16.46	6974	16.59	
Q2: 16501–19200	8446	20.03	8061	19.07	7946	18.90	
Q1: 911–16500	7907	18.75	7209	17.05	6842	16.27	
Mean ± SD (NTD)	26158	14791	25260	14316	24636	14682	<0.001
**Infections**							
Gingivitis	11852	28.07	5252	12.41	--	--	<0.001
Periodontitis	33327	78.94	14776	34.90	--	--	<0.001
Urinary tract infection	NA	NA	5032	11.89	--	--	--
Bacterial vaginosis	NA	NA	19704	46.54	--	--	--
Infections of genitourinary tract	NA	NA	522	1.23	--	--	--
Infections of kidney	NA	NA	619	1.46	--	--	--
Inflammatory disease of cervix, vagina and vulva	NA	NA	31032	73.30	--	--	--

Note: PD = babies born to periodontal/gingival women; INF = babies born to women with selected inflammatory condition(s)*; Control = babies born to non-periodontal, gingival or inflammatory women; 33.8NTD = 1USD (2001 dollar)

* Inflammatory Conditions include: gingivitis, periodontitis, urinary tract infection, bacterial vaginosis, and infections of genitourinary tract, infections of kidney, inflammatory disease of cervix, vagina and vulva.

Abbreviations: SD: standard deviation; NTD: New Taiwan Dollars; USD: US Dollars; Q: quintile

**Table 2:** Comorbidities analysis of mothers and perinatal-infant physiology in the matched cohorts.

	PD		INF		Control		*p*-value
	N = 42,217		N = 42,334		N = 42,143		
	No.	%	No.	%	No.	%	
**Systemic illness**							
Diabetes mellitus	1095	2.59	1133	2.68	995	2.36	0.011
Hypertension & pre-eclampsia	527	1.25	577	1.36	529	1.26	0.25
Chronic renal disease	30	0.07	33	0.08	20	0.05	0.19
Chronic lung disease	461	1.09	471	1.11	340	0.81	<0.001
Smoking, alcohol, drug and substance abuse	117	0.28	112	0.26	69	0.16	0.001
Edema/renal disease	60	0.14	65	0.15	43	0.10	0.097
Anemia	306	0.72	294	0.69	276	0.65	0.47
**Localized to reproductive tract**							
Antepartum hemorrhage	4348	10.30	4717	11.14	3401	8.07	<0.001
Chorioamnionitis	81	0.19	95	0.22	52	0.12	0.002
Amniotic sac	780	1.85	839	1.98	565	1.34	<0.001
Cervical incompetence	119	0.28	141	0.33	76	0.18	<0.001
Structural abnormality	214	0.51	215	0.51	174	0.41	0.071
**High risk pregnancy and others**							
Pregnancy with history of abortions	71	0.17	65	0.15	61	0.14	0.68
Pregnancy with other poor obstetric history	546	1.29	532	1.26	414	0.98	<0.001
Pregnancy with poor reproductive history	4	0.01	4	0.01	4	0.01	1.00
**Perinatal physiology**							
Preterm or low birth weight	1114	2.64	1175	2.78	1111	2.64	0.36
Congenital anomalies	7358	17.43	7226	17.07	6500	15.42	<0.001
Infections (specific)	3486	8.26	3468	8.19	3174	7.53	<0.001
Endocrine and metabolic disturbances	292	0.69	311	0.73	283	0.67	0.53
Hematological disorders	115	0.27	146	0.34	110	0.26	0.05
**Comorbidity in infancy**							
Accidental injuries	23912	56.64	23854	56.35	21776	51.67	<0.001
Burns	4567	10.82	4475	10.57	3995	9.48	<0.001
Pneumonia or influenza	31616	74.89	31677	74.83	30248	71.77	<0.001
Fever	28039	66.42	28113	66.41	26940	63.93	<0.001
Antibiotic poisoning	11	0.03	11	0.03	10	0.02	0.97

Note: PD = babies born to periodontal/gingival women; INF = babies born to women with selected inflammatory condition(s); Control = babies born to non-periodontal, gingival or inflammatory women

Multivariate survival analyses used include crude and adjusted Cox proportional hazards regression models which take into account premature deaths (including cases of sudden infant death syndrome) as competing risks. The survival models account for censoring among those who do not have an event during observation or are lost-to-follow-up. No model assumptions were violated. Effects of maternal age at delivery, residential area, parental income, baby sex, maternal comorbidities, history of high-risk pregnancy, perinatal physiology (preterm low birth weight, congenital anomalies, hematological disorders, or other specific infections), and comorbidity in infancy (fever, antibiotic poisoning, pneumonia or influenza, accidental injuries, or burns) are considered in the adjusted models if they appear statistically different (p<0.10) in the univariate analysis. All data management and hazard ratio calculations were done using the Statistical Analysis System (SAS) software for Windows (version 9.3; SAS Institute, Cary, NC).

## Results

For our study, a total of 42,217 singleton, livebirth babies born to periodontal or gingival women were identified in 2001 which formed the PD cohort. After frequency matching, the INF and control cohorts consisted of 42,334 and 42,143 matched singleton, livebirth babies born to mothers with selected inflammatory conditions and those born to mothers who were free from inflammation during pregnancy, respectively. Proportion of censoring followed a similar pattern and was not significantly different across the three groups (S4 Table).

Distributions of baseline socio-demographic variables for the matched cohorts were presented in our descriptive analysis in Table 1. Due to matching, distributions of baby sex and mother’s age at delivery were alike across the three cohorts. However, the proportion of mothers living in south and east Taiwan was significantly lower in PD when compared to INF and Control. Also, significantly more babies born to periodontal mothers were classified in the bottom income quintiles than babies born to non-inflammatory mothers: Q1 (18.75% vs. 16.27%) and Q2 (20.03% vs. 18.90%). Frequencies of periodontitis and gingivitis in PD were 78.94% and 28.07%, respectively.

Systemic and localized comorbidities of mothers, as well as history of high-risk pregnancy are illustrated in Table 2. We detected higher percentage of mothers with diabetes mellitus (p = 0.011), chronic lung diseases (p<0.001) and smoking, alcohol, drug and substance abuse (p = 0.001) in PD and INF cohorts relative to Control. Higher proportions of mothers with gum or selected infections were also found to have conditions localized to the reproductive tract such as antepartum hemorrhage (p<0.001), chorioamnionitis (p = 0.002), amniotic sac (p<0.001), cervical incompetence (p<0.001), and pregnancy with poor obstetric history (p<0.001). Prevalence of women with history of abortions, however, was not statistically different across cohorts. In perinatal and infant physiology, congenital anomalies, other specific infections, accidental injuries and burns, pneumonia or influenza, and fever were all statistically different.

**Fig 2:**
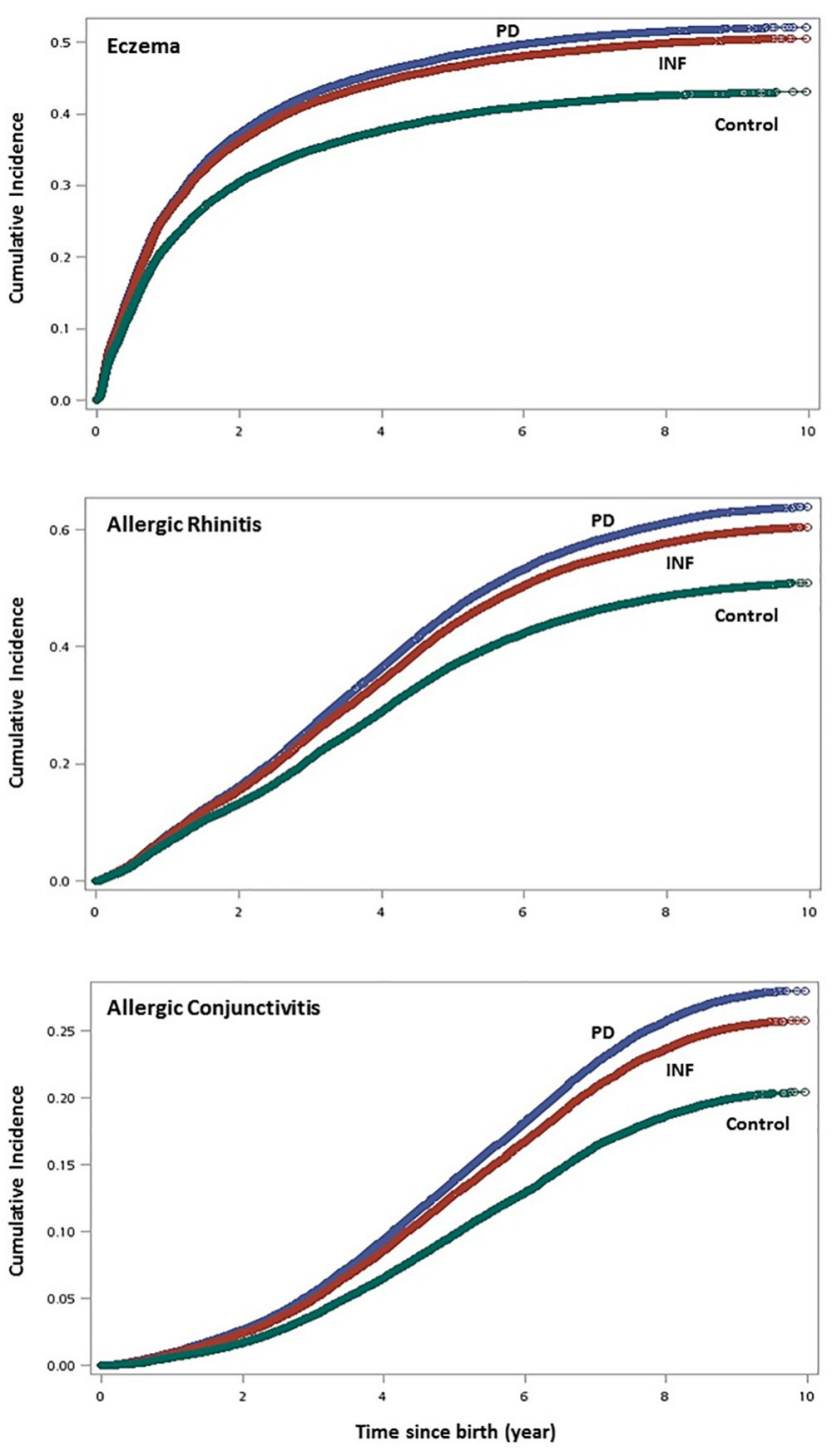
Cumulative incidence of childhood allergies using Kaplan-Meier survival curves. (top graph: eczema; middle: allergic rhinitis; bottom: allergic conjunctivitis).

Cumulative incidence of AR, AC and eczema were significantly higher in the inflammatory cohorts (PD and INF) compared with matched control as shown in the Kaplan-Meier curves (Fig 2). PD exhibited the highest cumulative incidence in the three types of child allergies: its 1-year cumulative incidences for AR, AC, and eczema were 7.5%, 0.95%, and 23.3%, respectively (vs. 7.3%, 0.88%, and 23.1% in INF, and 6.3%, 0.59%, and 19.6% in Control). Similarly, its respective 9-year cumulative incidences were 46.8%, 24.2%, and 40.4% (vs. 44.9%, 22.4%, and 39.4% in INF, and 39.5%, 18.3%, and 34.8% in Control). In addition, temporal progression of the three atopic diseases appear to resemble to that suggested by the atopic march theory which validated our method of identifying events: we observed a steady rate of onset for AR which persisted until the age of 6–7, while eczema had an apparent onset before the first 12 months of life and continued, although at a slower pace, into childhood.

**Fig 3:**
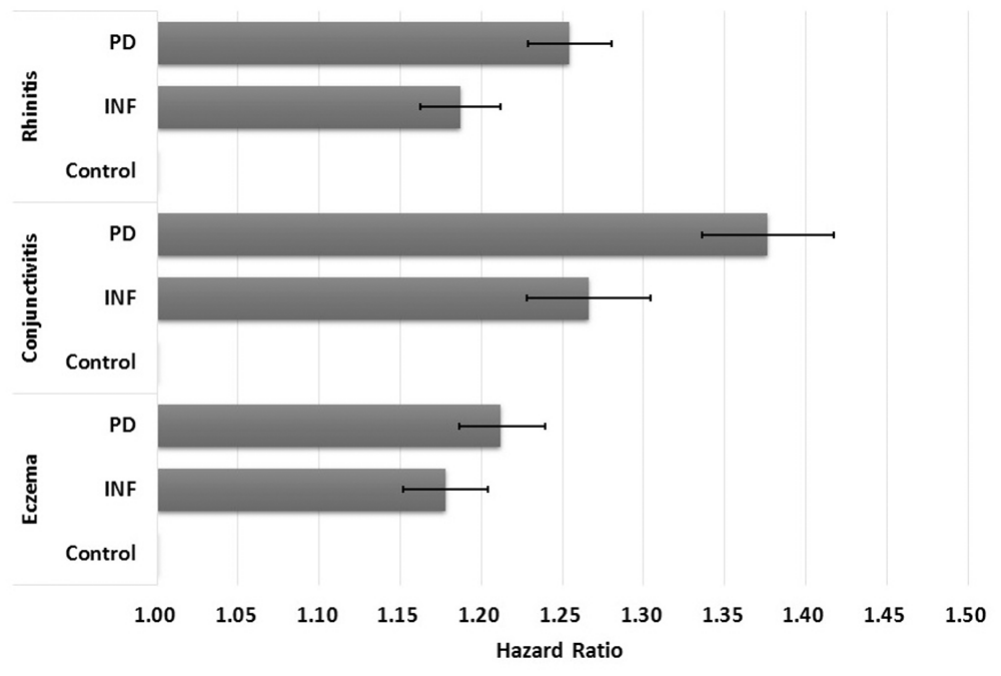
Crude hazard ratios and 95% confidence intervals for allergic rhinitis, allergic conjunctivitis, and eczema in children by study cohort. (PD—babies born to periodontal or gingival mothers; INF—babies born to mothers with selected inflammatory conditions; Control—babies born to non-inflammatory mothers).

Using crude multivariate survival analysis, the risk of developing AR, AC, and eczema in PD children compared to its matching control had crude hazard ratios (HRs) of 1.25 (95% confidence interval (CI): 1.23–1.28), 1.38 (1.34–1.42), and 1.21 (1.19–1.24), respectively (Fig 3). Similarly, children born to mothers with selected infections (INF) had moderately higher risk of developing these allergies: AR (HR: 1.19, 95% CI: 1.16–1.21); AC (HR: 1.27, 95% CI: 1.23–1.30); and eczema (HR: 1.18, 95% CI: 1.10–1.15). After adjusting for maternal age at delivery, mother’s residential area, parental income, baby sex, maternal comorbidities, history of high-risk pregnancy, perinatal physiology, and comorbidity in infancy, the risk of developing AR, AC, and eczema in PD children compared to its matching control had adjusted HRs of 1.17 (95% CI: 1.15–1.20), 1.27 (95% CI: 1.24–1.31), and 1.14 (95% CI: 1.12–1.17) (Table 3). INF children were also found to have adjusted HRs of 1.13 (95% CI: 1.11–1.15), 1.20 (95% CI: 1.16–1.24), and 1.12 (95% CI: 1.10–1.15) for developing AR, AC, and eczema, respectively.

**Table 3:** Predicting childhood incidence of the allergic rhinitis, allergic conjunctivitis, and eczema using Cox proportional hazards models with adjusted hazard ratios (aHRs) and 95% confidence intervals.

	Allergic Rhinitis			Allergic Conjunctivitis			Eczema		
	aHR	95%CI	*p*-value	aHR	95%CI	*p*-value	aHR	95%CI	*p*-value
**Cohort**									
PD	**1.17**	1.15–1.20	<0.001	**1.27**	1.24–1.31	<0.001	**1.14**	1.12–1.17	<0.001
INF	**1.13**	1.11–1.15	<0.001	**1.20**	1.16–1.24	<0.001	**1.12**	1.10–1.15	<0.001
Control	**1**	--	--	**1**	--	--	**1**	--	--
**Maternal age (years)**									
	**1.01**	1.01–1.02	<0.001	**1.03**	1.02–1.03	<0.001	**1.00**	1.00–1.00	0.01
**Baby sex**									
Female	**0.79**	0.78–0.80	<0.001	**0.84**	0.82–0.86	<0.001	**0.99**	0.97–1.01	0.20
Male	**1**	--	--	**1**	--	--	**1**	--	--
**Maternal comorbidity (vs. no)**									
Diabetes mellitus	**1.15**	1.09–1.21	<0.001	**1.14**	1.07–1.22	<0.001	**1.10**	1.05–1.17	<0.001
Chronic lung disease	**1.34**	1.25–1.45	<0.001	**0.95**	0.85–1.07	0.41	**1.09**	1.00–1.19	0.04
Edema/Renal disease	**0.92**	0.74–1.13	0.41	**0.94**	0.70–1.27	0.69	**1.07**	0.85–1.35	0.55
Smoking, alcohol, drug and substance abuse	**1.21**	1.03–1.41	0.02	**1.20**	0.96–1.49	0.11	**1.21**	1.02–1.43	0.03
Antepartum hemorrhage	**1.02**	0.99–1.05	0.12	**1.04**	1.00–1.08	0.07	**1.04**	1.01–1.07	0.01
Chorioamnionitis	**1.17**	0.97–1.40	0.11	**0.84**	0.62–1.13	0.25	**0.87**	0.69–1.08	0.20
Amniotic sac	**0.99**	0.93–1.05	0.71	**1.13**	1.04–1.23	0.005	**1.04**	0.97–1.11	0.28
Cervical incompetence	**1.04**	0.89–1.22	0.60	**0.84**	0.66–1.07	0.16	**1.12**	0.95–1.32	0.19
Structural abnormality	**1.05**	0.93–1.17	0.43	**1.09**	0.93–1.27	0.28	**1.15**	1.01–1.30	0.02
Pregnancy with other poor obstetric history	**1.10**	1.02–1.19	0.01	**1.22**	1.10–1.35	<0.001	**1.48**	1.37–1.59	<0.001

Note: PD = babies born to periodontal/gingival women; INF = babies born to women with selected inflammatory condition(s); Control = babies born to non-periodontal, gingival or inflammatory women. Model adjusted for maternal age at delivery, residential area, parental income, baby sex, maternal comorbidities, history of high-risk pregnancy, perinatal physiology, and comorbidity in infancy. Abbreviations: aHR = adjusted hazard ratio; CI = confidence interval.

## Discussion

There is evidence indicating that prenatal exposure to bacteria may prompt *in utero* sensitization and exaggerate the immune activity of the developing fetus. Maternal exposure to pathogens, particularly during pregnancy, which activates the immune-inflammatory cascade can potentially alter fetal programming of antibody production and allergy response through CB-IgE [13–15,26]. Typically, eczema is the first atopic manifestation as a consequence of early sensitization by CB-IgE. According to the atopic march theory, it is a predictor for subsequent development of allergic respiratory and ocular diseases during childhood [19]. In the current study, we find the overall cumulative incidence and risks of children born to periodontal or inflammatory pregnant mothers for AR, AC, and eczema are moderately but significantly higher than those born to non-inflammatory counterparts. Infection, particularly gum infection, in pregnant women is thus considered an independent risk factor for these three allergic diseases in children.

Early life onset of allergic diseases can have a serious developmental and psychosocial impact on children. To our knowledge, this is one of the first epidemiological studies to examine intergenerational associations between maternal periodontal diseases and the development of AR, AC, and eczema in children. Previous research primarily focus on the development of allergy by examining immune responses to oral bacteria and immunologic pathogenesis of periodontal diseases in either children or adults [27–30]. In a national survey study of 9,385 subjects above 12 years of age, elevated immune response to periodontal bacteria through higher IgG antibody concentrations is associated with lowered risk of allergic diseases [31]. Another study in adults similarly found an inverse relationship between respiratory allergies and periodontitis which was explained in part by the hygiene hypothesis [27]. Other studies have solely explored the prevalence and incidence of childhood allergies under different geographical and cultural context [1–2,25,32].

We exploited data from a nationwide database with a longitudinal design in order to causally decipher the link between maternal gum inflammation and the development of allergic diseases in children. Unlike past survey research, we have a larger, population-based sample. More importantly, our research features the intergenerational relay of infection from mothers to children. Also, we do not have the problem of parental recall bias when details about periodontal and overall health during pregnancy need to be surveyed for study. Since periodontal treatments, or simply general dental visits, are typically avoided during pregnancy in the current population [11], it is expected that we have identified mothers with chronic inflammations by extracting subjects within this time window (and not mothers with asymptomatic or acute periodontal disease who are unlikely to seek dental care during pregnancy). Premature deaths, which can bias our results, are considered as competing risk in the survival analyses. Additionally, the confounding effect of maternal comorbidities, poor obstetric history, antibiotic use and perinatal and infant illnesses has been controlled.

Nevertheless, there are several limitations to our study. First, we cannot retrieve information on the type of food consumption for these studied mothers during their pregnancy, or the living conditions in which they may expose themselves to allergens such as pollen or dust mites during this period of time. We tried to control this confounding effect by adjusting for residential area and parental income in multivariate analyses. Likewise, whether the babies are breastfed or the type of delivery performed cannot be identified using the claims-based insurance database. Additionally, severity of the periodontal diseases cannot be classified in the mothers. This may generate variable influence on the fetal immune development. Family history of allergies which correlates with gene predisposition to allergies is not being explored because it is beyond the scope of this study. However, risk of developing atopy has been shown to be higher in children with atopic heredity and genetic predisposition [18,33–34]. Family history of atopy seems to affect development of allergic diseases through elevated CB-IgE or early-life sensitization by aero- or food allergens [18]. The filaggrin gene is also a strong predisposing factor as its mutations leads to defective epithelial skin barrier and the initiation of the atopic march [34]. Finally, we may have underestimated the prevalence of periodontitis given the claims-nature of our data. This is because the NHI reimbursement criteria is less sensitive to capture true cases as it uses a higher pocket depth threshold when compared with the Centers for Disease Control and Prevention-the American Academy of Pediatrics (CDC-AAP) case definition; mild cases should be considered to provide a comprehensive picture of periodontal epidemiology [35].

Although these allergic conditions may not be life-threatening and detrimental to children’s health, but they are highly prevalent among children in modern society and can greatly affect the quality of their daily living and generate a substantial societal cost through their lifetime. Periodontal care should thus be actively promoted during pregnancy. The exact mechanisms in which prenatal exposure to oral pathogens and its subsequent activation of the inflammatory cascade (which alters immune response of the offspring) may act via various pathways and require further investigation.

In summary, we adopted a population-based, retrospective cohort study with a nine-year follow-up period to assess the risk of developing childhood allergic rhinitis, allergic conjunctivitis, and eczema in pregnant women with periodontal diseases from Taiwan. Our results suggest significantly higher incidences and risks of developing these allergic diseases for children born to mothers with gum and other inflammation in pregnancy compared to those born to inflammation-free mothers.

## Supporting Information

S1 Table ICD-9-CM codes used for identification of infections and inflammatory conditions in mothers. (DOCX)

S2 Table ICD-9-CM codes used for identification of events. (DOCX)

S3 Table Names and ICD-9-CM codes used for identification of maternal comorbidities, perinatal conditions, and comorbidities in infancy. (DOCX)

S4 Table Proportion of censoring in PD, INF and control groups for the three events under study. (DOCX)
