# Supplementary material for: Risk of Allergic Rhinitis, Allergic Conjunctivitis, and Eczema in Children Born to Mothers with Gum Inflammation during Pregnancy
Source: PLoS One. 2016 May 25;11(5):e0156185. doi: 10.1371/journal.pone.0156185 (PMC4880316; doi:10.1371/journal.pone.0156185)
Supplement: S1 Table — (DOCX) [file pone.0156185.s001.docx]

**Supplementary Materials**

**Supplementary Table 1. ICD-9-CM codes used for identification of infections and inflammatory conditions in mothers.**

| **Infections** | **ICD-9-CM code** |
| --- | --- |
| Gingivitis | 523.0-523.2 |
| Periodontitis | 523.3-523.5 |
| Urinary tract infection | 599.0 |
| Bacterial vaginosis | 616.1 |
| Infections of genitourinary tract | 646.6, A39.2 |
| Infections of kidney | 590 |
| Inflammatory disease of cervix, vagina, and vulva | 616 |
