# Supplementary material for: Risk of Allergic Rhinitis, Allergic Conjunctivitis, and Eczema in Children Born to Mothers with Gum Inflammation during Pregnancy
Source: PLoS One. 2016 May 25;11(5):e0156185. doi: 10.1371/journal.pone.0156185 (PMC4880316; doi:10.1371/journal.pone.0156185)
Supplement: S2 Table — (DOCX) [file pone.0156185.s002.docx]

**Supplementary Materials**

**Supplementary Table 2. ICD-9-CM codes used for identification of events**

We identified our events using ICD-9-CM classification. The codes used are listed in the following:

| **Event** | **ICD-9-CM code^a^** |
| --- | --- |
| Allergic rhinitis (incl. hayfever) | 477 |
| Allergic conjunctivitis | 372.14 |
| Eczema | 692 |

^a^ International Classification of Diseases, Ninth Revision, Clinical Modification (ICD-9-CM), Sixth Edition.
