# Supplementary material for: Risk of Allergic Rhinitis, Allergic Conjunctivitis, and Eczema in Children Born to Mothers with Gum Inflammation during Pregnancy
Source: PLoS One. 2016 May 25;11(5):e0156185. doi: 10.1371/journal.pone.0156185 (PMC4880316; doi:10.1371/journal.pone.0156185)
Supplement: S3 Table — (DOCX) [file pone.0156185.s003.docx]

**Supplementary Materials**

**Supplementary Table 3. Names and ICD-9-CM codes used for identification of confounding variables including maternal comorbidities, perinatal conditions, and comorbidities in infancy.**

| **Name** | **ICD-9-CM code** |
| --- | --- |
| **Maternal Comorbidity** |  |
| Systemic |  |
| - Diabetes mellitus | 250, 648.0, 648.8, A18.1 |
| - Chronic lung disease | 490-492, 496, A32.3, A32.5 |
| - Edema/Renal disease | 646.1-646.2 |
| - Smoking, alcohol, drug and substance use | 291, 303-304, 386, 648.3, 649.0, A21.6 |
| Localized to reproductive tract |  |
| - Antepartum hemorrhage, abruptio placentae, and placenta previa | 641 |
| - Chorioamnionitis | 658.4 |
| - Amniotic cavity and membranes | 657, 658.0, 658.8-658.9 |
| - Cervical incompetence | 654.5 |
| - Structural abnormality (uterus/cervix/vagina/vulva) | 654.0-654.1, 654.3, 654.4, 654.6-654.9 |
| High-risk pregnancy |  |
| - Pregnancy with other poor obstetric history (inc. preterm labor history) | V23.4 |
| **Perinatal Conditions** |  |
| - Preterm or low birth weight | <37 weeks or <2500g; 764, 765, A45, V21.3 |
| - Congenital anomalies | 740-759, A44 |
| - Infections (specific) | 771 |
| - Hematological disorders | 776 |
| - Other conditions | 778-779 |
| **Comorbidity in Infancy** |  |
| - Accidental injuries | 910-929, A55, E50 |
| - Burns | 940-949, A52, E51 |
| - Gingivitis | 523.0-523.2 |
| - Periodontitis | 523.3-523.5 |
| - Pneumonia or Influenza | 481-487 |
| - Fever (in 1st year after birth) | 780.6, A460 |
