# Supplementary material for: Risk of Allergic Rhinitis, Allergic Conjunctivitis, and Eczema in Children Born to Mothers with Gum Inflammation during Pregnancy
Source: PLoS One. 2016 May 25;11(5):e0156185. doi: 10.1371/journal.pone.0156185 (PMC4880316; doi:10.1371/journal.pone.0156185)
Supplement: S4 Table — (DOCX) [file pone.0156185.s004.docx]

**Supplementary Materials**

**Supplementary Table 4. Proportion of censoring in PD, INF and control groups for the three events under study.**

| **Proportion of censoring (%)** | **PD** | **INF** | **Control** |
| --- | --- | --- | --- |
| AR | 53.2 | 55.1 | 60.5 |
| AC | 75.8 | 77.8 | 81.7 |
| Eczema | 59.6 | 60.6 | 65.2 |
